# Transverse testicular ectopia with Müllerian duct remnant in an incarcerated congenital inguinal hernia – a case report

**DOI:** 10.1186/s12894-020-00680-9

**Published:** 2020-07-30

**Authors:** Mostafa Kotb, Ahmed Hassan, Mohamed Abouheba

**Affiliations:** Pediatric Surgery, Alexandria Faculty of Medicine, Alexandria, Egypt

**Keywords:** Case report, Incarcerated hernia, Müllerian duct, testicular ectopia

## Abstract

**Background:**

Transverse testicular ectopia (TTE) is a rare anomaly characterized by both testes descending through a single inguinal canal. In this report, the diagnosis of TTE was discovered in the event of an incarcerated congenital inguinal hernia in a neonate.

**Case presentation:**

We present a case of TTE accompanied by persistent Müllerian duct structures (PMDS) that had been discovered incidentally during inguinal exploration of a 26-day-old boy who presented with an incarcerated congenital inguinal hernia on the right side and left cryptochidism on the left side. The pathogenesis, approach and proposed management of TTE are discussed.

**Conclusion:**

TTE is an extremely rare anomaly, especially in neonates, and should be suspected in patients presenting with inguinal hernia on one side and cryptorchidism on the other side.

## Background

Transverse testicular ectopia (TTE), also named testicular pseudo-duplication or crossed testicular ectopia, is a rare anomaly characterized by both testes descending through a single inguinal canal [1]. Most of the cases (65%) are diagnosed intraoperatively during an inguinal hernia repair, while a few are diagnosed preoperatively [2]. We present a case of TTE accompanied by persistent Müllerian duct structures (PMDS) that had been discovered incidentally during inguinal exploration of a male neonate presenting with an incarcerated congenital inguinal hernia on the right side and left cryptochidism on the left side. The pathogenesis, approach and proposed management of TTE were discussed.

## Case presentation

**Fig. 1:**
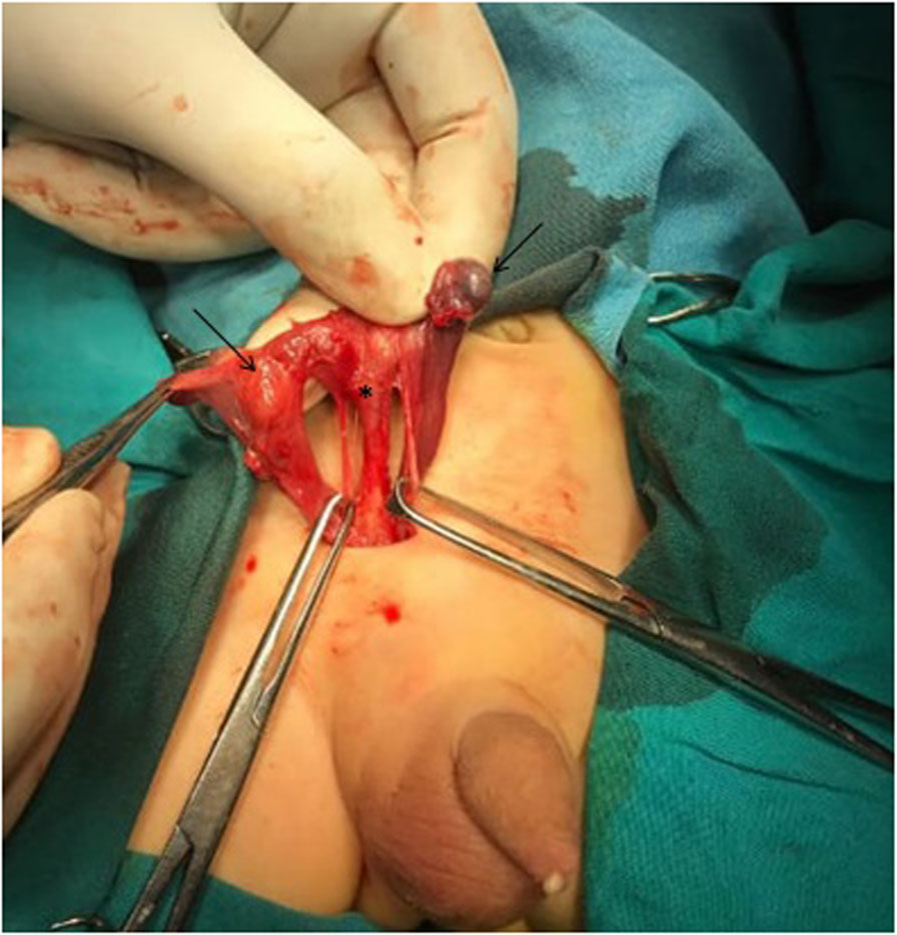
Introperative photo showing both testes (arrows) on the right side and rudimentary uterus (asterisk)

A 26-day-old boy was referred from a peripheral hospital with a right-sided incarcerated congenital inguinal hernia. The referring pediatrician told his father that he has a palpable testis in his right inguinal canal but no palpable testis on the left side. Apart from a clinically evident right irreducible hernia and empty both hemiscrota, the rest of the examination was unremarkable. After initial resuscitation with intravenous fluids and antibiotics, an urgent operation was undertaken. On inguinal exploration, the hernia sac was found to contain a viable cecum and small bowel loops. After reduction of the contents to the peritoneal cavity, the right testis was found in the inguinal canal. However, a second testis was unexpectedly delivered through the deep inguinal ring. Both testes have independent spermatic cords, i.e. two separate sets of vas deferens and testicular vessels on either side of a T- shaped structure resembling an infantile uterus and fallopian tube (Fig. 1). Because of the shortness of the spermatic cord and the vague nature of this anomaly for the operating surgeon, only a biopsy was taken from both testes and all the structures were returned back to the peritoneal cavity followed by herniotomy.

Postoperative pathology showed normal testicular tissue bilaterally with absent ovarian structures. Karyotyping was done for the patient and revealed a normal 46XY male karyotype. Diagnostic laparoscopy was performed at the age of 6 months, which showed a closed left internal ring on the left side and a widely open internal ring on the right side. In addition, the left sided testis and spermatic cord were found joining their counterparts on the right side through the rudimentary uterus close to the right internal ring. The rudimentary uterus as well as the fallopian tubes were partially excised. Peritoneal dissection was made to gain extra length for both spermatic cords. Orchiopexy was done, with each testis fixed into its corresponding hemiscrotum. The patient had an uneventful recovery. Throughout the 6-month follow-up period, the size and the blood flow of both testes were normal as evaluated by Doppler ultrasound.

## Discussion and conclusions

TTE is a rare entity that usually presents in childhood, with a mean age of 4 years [3]. To our best knowledge, the present case represents the first TTE diagnosed in a neonate with irreducible inguinal hernia. The precise etiology of TTE is still unclear. Nevertheless, various anatomic factors (for instance, tearing or rupture or defective implantation of the gubernaculum, deep inguinal ring occlusion, adhesions developing between the testis and adjacent structures, late closure of the umbilical ring, etc.) are suggested to result in testicular maldescent [4].

TTE can be classified according to the presence of associated anomalies into 3 types; type 1 (associated with hernia only, 50% of the cases), type 2 (associated with PMDS, 30% of cases) and type 3 (associated with genitourinary abnormalities, 20% of cases) [5]. Our case belongs to type 2, which results from defects either in the synthesis or action of Müllerian inhibiting factor (MIF) and is characterized by the presence of uterus and fallopian tubes in otherwise phenotypically normal 46 XY males. PMDS is mostly discovered incidentally during surgery for inguinal hernia or cryptorchidism [6]. In patients with inguinal hernia and cryptorchidism, possible TTE and PMDS must be considered requiring radiologic evaluation of the genitourinary system and karyotyping [7].

Unfortunately, the overall occurrence of malignant transformation is 18%, which is nearly equal to that of abdominal testes in otherwise normal men [8]. Among the tumors which were reported with PMDS are seminoma, embryonal carcinoma, yolk sac tumor and teratoma. Moreover; these testes are subjected to a higher risk of malignant transformations arising from Müllerian remnants to squamous cell carcinoma, adenocarcinoma and papillary cystadenocarcinoma. Hence, long term follow-up for tumors is required [9].

The optimal surgical management of PMDS is still debatable. A staged procedure remains the most acceptable option as long as PMDS is usually found incidentally during surgery for inguinal hernia repair or undescended testis [10]. Testicular biopsies were taken and then replacing the testes, uterus, and fallopian tubes back through the deep inguinal ring to the abdominal cavity along with herniotomy are performed in the first stage. This is to be followed by the definitive surgery after confirming PMDS. Care should be taken as the vasa deferentia are usually found embedded in the wall of the uterus and the epididymis is contained in the mesosalpinx. Therefore, the removal of Müllerian remnants may injure these structures and interfere with the testicular blood supply as well [10]. Given the reported incidence of malignant transformation from the retained Müllerian structures along with their interference with the intrascrotal placement of the testes, as the free segment of the spermatic cord is usually short, partial removal of these structures without jeopardizing the vasa deferentia seems to be a valuable option. This was described by Guerrier et al. [11] that entails extensive dissection of the spermatic cord along with corporal hysterectomy, bilateral proximal salpingectomies while leaving fimbria and cervix attached to the epididymis to avoid vasa deferentia injury, and bilateral orchiopexy.

To sum up, TTE should be put in consideration whenever a patient presents with inguinal hernia on one side and undescended testis on the opposite side. Because of the risk of malignant transformation and interference with scrotal placement of the testis, MD remnants should be removed without jeopardizing the vas deferens, epididymis and testicular vessels in addition to herniotomy and orchiopexy. In addition, a long-term follow-up will be needed for assessment of malignant transformation and fertility in these patients.

## Data Availability

All data supporting the study are presented in the manuscript or available upon request.

## Abbreviations

MIF: Müllerian inhibiting factor
PMDS: Persistent Müllerian duct structures
TTE: Transverse testicular ectopia
